# Physiological Roles of β-amyloid in Regulating Synaptic Function: Implications for AD Pathophysiology

**DOI:** 10.1007/s12264-022-00985-9

**Published:** 2022-11-28

**Authors:** Wenwen Cai, Linxi Li, Shaoming Sang, Xiaoli Pan, Chunjiu Zhong

**Affiliations:** 1 Department of Neurology, Zhongshan Hospital, Fudan University, Shanghai, 200032 China; 2 Basic Medical College, Nanchang University, Nanchang, 330031 China; 3 State Key Laboratory of Medical Neurobiology, Institutes of Brain Science & Collaborative Innovation Center for Brain Science, Fudan University, Shanghai, 200032 China

**Keywords:** Aβ, Physiological role, Synapse, LTP, Synaptic vesicle cycle, Cognition, AD

## Abstract

The physiological functions of endogenous amyloid-β (Aβ), which plays important role in the pathology of Alzheimer's disease (AD), have not been paid enough attention. Here, we review the multiple physiological effects of Aβ, particularly in regulating synaptic transmission, and the possible mechanisms, in order to decipher the real characters of Aβ under both physiological and pathological conditions. Some worthy studies have shown that the deprivation of endogenous Aβ gives rise to synaptic dysfunction and cognitive deficiency, while the moderate elevation of this peptide enhances long term potentiation and leads to neuronal hyperexcitability. In this review, we provide a new view for understanding the role of Aβ in AD pathophysiology from the perspective of physiological meaning.

## Introduction

Alzheimer's disease (AD) is an irreversible neurodegenerative disorder and the most common cause of dementia [1, 2], which clinically manifests as progressive cognitive impairment and is pathologically characterized by extracellular amyloid-β (Aβ) plaques and intraneuronal neurofibrillary tangles [3]. Indisputable human genetic evidence and abundant data from biochemistry, histology, and animal models have established that Aβ is a key player in the pathogenesis of AD. However, along with a series of failures in clinical trials for the treatment and prevention of AD targeting Aβ [4, 5], there is a growing debate about its critical role in the pathogenesis of the disease.

**Fig. 1:**
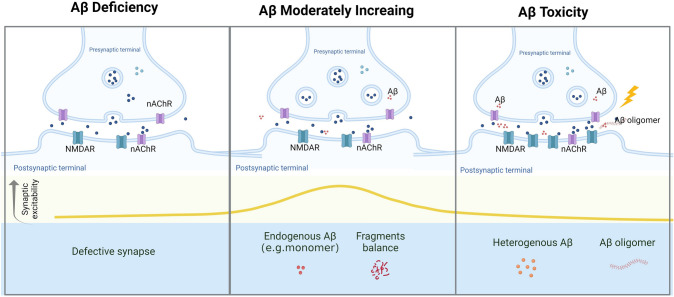
Schematic representation of the suggested physiological and pathological roles of Aβ in the synapse. Created with https://biorender.com/.

More than three decades have passed since Aβ was first identified in 1984 when Aβ was recognized as an endogenous neuropeptide that is physiologically metabolized in the central nervous system [6]. The Aβ sequence can be dated to ~ 500 million years ago, and the sequence homology in mammals exceeds 95% [7]. The conservation in evolution means that Aβ is critical to providing a selective advantage in the survival of species. Recently, accumulating studies have implied that Aβ plays roles in cognitive functions, synaptic functions, angiogenesis, antimicrobial response, tumor suppression, recovery from injury, and neurogenesis [8]. Especially, the roles at the synapse and antimicrobial role of Aβ [9–11], potentially explain the lack of efficacy and adverse effects in the clinical trials targeting Aβ production (Fig. 1).

The synapse is widely regarded as the basic biological structure of memory. As early as 1991, it was recognized that synaptic loss is a factor correlated with the cognitive deficit in AD [12] and an important cytopathological feature of cognitive decline [13]. It has been reported that Aβ regulates synaptic function in early AD [14, 15]. Given the pivotal role of the synapse in the mechanisms of learning and memory, elucidating how Aβ influences synaptic activity may benefit the understanding of AD pathology.

Here, we concentrate on evidence from research on the functions of Aβ in synaptic terminals. To begin with, several key points concerned with physiological conditions, which are usually omitted, will be elucidated. Next, the potential necessary and sufficient role of Aβ in synaptic function will be expanded into two parts (Table 1). The necessary role will be drawn from laboratory data in which Aβ itself was ablated or the generation pathway was blocked, mainly referring to amyloid precursor protein (APP) and BACE1 (β-site APP-cleaving enzyme 1). In contrast, the sufficient role will be discussed by underscoring the effect of moderately increased Aβ, but not toxic levels, on synaptic plasticity and neural excitability. Further, the underlying mechanism and several contradictions in these evidence will be listed. Last, we considered the physiological role of Aβ at the synapse in AD therapeutics and research on its pathology.

**Table 1:** Potential physiological roles of Aβ in the regulation of synaptic function.

Dosage of Aβ	Targeted Aβ type	Experimental paradigm	Effect	Year	References
Low (antibody)	Aβ, Aβ42	Conditional injection of 4G8 or exogenous Aβ42 by hippocampal cannula implant in mice	Injection of 4G8 disrupted short-term memory and long-term memory; memory consolidation induced by Aβ42 at the picomolar level	2009	[30]
Low (antibody)	Aβ, Aβ42	Blocking endogenous Aβ with monoclonal antibody JRF/rAb2	Impaired LTP in electrophysiology and cognitive deficits in behavior test, all of which were rescued by human Aβ 42 (200 pmol/L)	2011	[31]
Low (siRNA)	Aβ	siRNA against murine APP	Attenuation of LTP		
Null (genetic depletion)	Aβ	APP null mice	Reactive astrogliosis after 14 weeks; 15–20% body weight loss and decreased forelimb grip	1995	[32]
Null (genetic depletion)	Aβ	APP null mice	Age-dependent cognitive impairment, LTP impairment, synaptic loss	1999	[33]
Null (genetic depletion)	Aβ	APP null mice	Decreased dendritic length and projections in CA1 neurons; LTP deficiency associated with PTP	1999	[34]
Null (genetic depletion)	Aβ	Primary hippocampal neurons from APP KO mice	Restricted neurite outgrowth, reduced neuronal branches, and shortened axons; enhanced cellular adhesion	2019	[35]
Null (genetic depletion)	Aβ, oAβ42	Hippocampal slices; APP KO mice	Alteration of neurotransmitter release, LTP and synaptic ultrastructure under oAβ42 at picomolar concentrations	2019	[36]
Low (genetic depletion)	Aβ	Selective inactivation of APP/APLP1/APLP2 in excitatory neurons	Impaired synaptic plasticity, learning and memory; neuronal hyperexcitability	2020	[37]
Null (genetic depletion)	Aβ	*BACE1*^–/–^mice; m/h-APPswe; hPS1ΔE9 transgenic mice	Low anxiety; synaptic plasticity impairment; cognitive deficits in behavior test, rescued by APP/PS1 hybridization	2005	[38]
Null (genetic depletion)	Aβ	Tamoxifen induced conditional knockout of BACE1 in adult mice	CA1 LTP damage	2019	[39]
Null (genetic depletion)	Aβ	*BACE1*^–/–^mice	Synaptic disorders in hippocampal CA3 pyramidal neurons	2014	[40]
Null (genetic depletion)	Aβ	*BACE1 *^–/–^mice	LTP deficiency in mossy fiber transmission to hippocampal CA3 synapses	2008	[41]
Null (genetic depletion)	Aβ	*BACE1*^–/–^mice	Motor-sensory disturbances, spatial memory deficits, and seizures	2008	[37]
Null (genetic depletion)	Aβ	*BACE1*^*fl/fl*^ mice	LTP damage, amyloid deposits reduced	2018	[42]
Null (genetic depletion)	Aβ	Hippocampal slices from *BACE1*^–/–^mice	Severe presynaptic defect at mossy fiber to CA3 neurons, rescued by nAChR agonists	2010	[43]
Low (pharmacological inhibition)	Aβ	Oral administration of BACE1 inhibitor SCH1682496 or LY2811376 to mice	Reduced spine formation in layer V pyramidal neurons, impaired mEPSCs and LTP	2015	[44]
Low (pharmacological inhibition)	Aβ	Intracranial injection of the γ-/β-secretase inhibitor into rats	Consolidation of fear memory	2020	[45]
moderately increase	Aβ42	PS1 and PS2 conditional double KO mice, 3XTg-AD mice	Ventricular infusion of Aβ1-42 monomers improved the impaired memory	2022	[46]
200 pmol/L/nmol/L	oAβ40, oAβ42	Hippocampal slices; injection Aβ in hippocampus	At 200 nmol/L, oAβ40, oAβ42 and monomeric Aβ42 impaired LTP and cognitive function, while only oAβ42 at 200 pmol/L enhanced synaptic plasticity and memory function; 200 pmol/L of oAβ42 rescued LTP impairment induced by murine Aβ antibody	2018	[47]
Picomolar	Aβ42	Primary hippocampal neurons; injection of Aβ42 in hippocampus	Short-term exposure to Aβ42 enhanced LTP and cognition but long-term exposure impaired it	2016	[48]
Gradient	Aβ42	Hippocampal slices; injection of Aβ42 in hippocampus	Aβ42 dose-dependent alteration of LTP and behavior performance	2012	[49]
Picomolar	Aβ42	Hippocampal slices; injection of Aβ42 in hippocampus	Enhanced LTP and cognitive function in behavior test under picomolar Aβ42	2008	[50]
Physiological	Aβ	Acute brain slices	Increased synaptic activity promoted Aβ release	2005	[51]
Physiological	Aβ	Aβ microdialysis and EEG *in vivo*	Increased synaptic activity promoted Aβ release	2008	[52]
Moderately elevated	Aβ	Primary rat neurons	Moderately elevated Aβ resulted in increased SV recycling at both excitatory and inhibitory synapses	2017	[53]
200 pmol/L	Aβ1-16	Primary rat neurons	Aβ1-16 but not Aβ17-42 increased SV recycling at glutamatergic synapses	2021	[54]
Moderately elevated	Aβ	Primary hippocampal neurons; hippocampal slices	Both increasing and decreasing endogenous Aβ attenuated short-term facilitation in excitatory synaptic connections	2009	[55]
pmol/L–nmol/L	Aβ10-15	5XFAD and APP/PS1 mice; hippocampal slices	N-terminal Aβ fragment containing Aβ10-15 reversed synaptic dysfunction in 5XFAD and APP/PS1 mice	2021	[56]
pmol/L–nmol/L	Aβ10-15	N2A cell line; primary hippocampal neurons	N-terminal Aβ fragment containing Aβ10-15 reversed Aβ-induced neuronal toxicity	2018	[57]
pmol/L–nmol/L	Aβ10-15	N2A cell line; primary hippocampal neurons; injection in hippocampus	N-terminal Aβ fragment containing Aβ10-15 enhanced LTP and conditional fear memory	2014	[58]
100 μmol/L	Aβ oligomers	Primary hippocampal neurons; hippocampal slices	Aβ oligomers increased dendritic complexity and dendritic spine density	2020	[59]

LTP, Long-term potentiation; PTP, Post-tetanic potentiation; KO, Knockout; oAβ42, oligomeric Aβ42; APLP 1/2, amyloid precursor-like protein 1/2; mEPSC, miniature excitatory postsynaptic current; nAChR, nicotinic acetylcholine receptor; nmol/L/pmol/L, nano-/pico-molar; EEG, electroencephalogram; SV, Synaptic vesicle

## Keys Concerns with Physiological Aβ

### Biogenesis and Metabolism

**Fig. 2:**
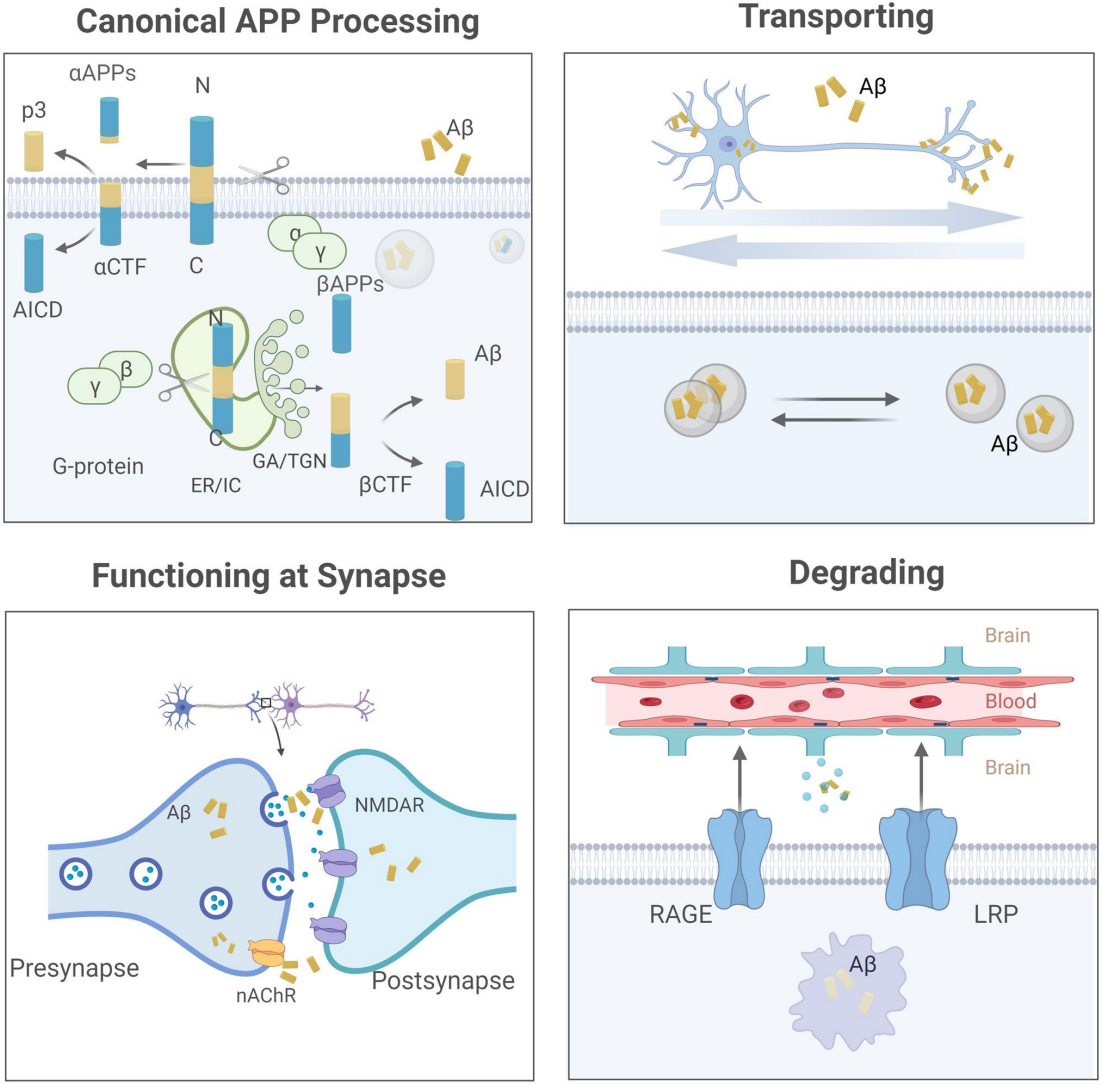
Schematic representation of the biogenesis and metabolism of Aβ. (1) Canonical APP processing. APP inserted on the cellular membrane is cleaved by α-secretase in an amyloidogenic manner, and internalized APP is proteolyzed by β-secretase in subcellular compartments to produce Aβ; (2) Transporting. Aβ along with CTF is packaged into vesicles or is secreted into extracellular space, and Aβ can be transported intracellularly in both anterograde and retrograde directions; (3) Functioning at synapse. Aβ performs the function in the intra- and extra-cellular space, and the presynaptic nicotinic acetylcholine receptor (nAChR) mediates Aβ reuptake at the synaptic terminal; (4) Degrading. Aβ is transported by lipoprotein receptor-related protein (LRP) and receptor for advanced glycation end products (RAGE). In cells, Aβ can be degraded by insulin-degrading enzymes and neprilysin or be bound by peripheral substances. Created with https://biorender.com/.

APP is encoded by 19 exons on the long arm of chromosome 21, of which exons 16 and 17 are responsible for encoding Aβ. APP family proteins are type I single-pass transmembrane proteins; the other two isoforms, amyloid precursor-like proteins 1 and 2 (APLP1/2) cannot produce Aβ peptide. According to the splicing sequence, APP695, APP751, and APP770 have been described most often, and APP695 is the main isoform in the human brain. See the biosynthesis and metabolic fate of Aβ in Figure 2.

Although Aβ is generated from APP in a complex manner, canonical processing by α/β/γ-secretase is dominant, including an amyloidogenic and a non-amyloidogenic pathway. The former pathway happens in subcellular compartments like endoplasmic reticulum/intermediate compartment, and Golgi apparatus/trans-Golgi network [16–18], where internalized APP is proteolyzed by β-secretase on the 671–672 amino-acid sequence [19], exposing the N-terminus of Aβ, and then γ-secretase works to generate the C-terminus, forming a chain with 37–49 amino-acids named Aβ. Generally, Aβ40 (~ 90% of total Aβ) and Aβ42 (~ 5%–10% of total Aβ) are predominant [20, 21], and Aβ42 is more prone to deposition than Aβ40 due to the strong hydrophobicity of the C-terminal amino-acid residue. The unhydrolyzed APP is located at the cell surface and is processed by the latter means, in which α-secretase cleaves at amino-acids 16–17 on the Aβ sequence to generate a soluble fragment αAPPs and α C‑terminal fragments, which are further catalyzed by γ‑secretase to generate p3 [22, 23].

The mature Aβ along with the C-terminal fragment (CTF) is packaged into vesicles or is secreted into extracellular space. Intracellularly, Aβ can be transported in both anterograde and retrograde directions. APP [24] as well as somatic Aβ [25] are transported in the fast anterograde component, while retrograde transport to cell bodies occurs when Aβ is absorbed by synaptic reuptake or is produced by APP internalized from distal axon terminals [26]. Besides, transport of the compartment containing BACE and PS1 requires APP, which may function as a kinesin-I membrane receptor [27]. After performing its function in the intra- and extra-cellular space (see below), Aβ under physiological conditions maintains a balance that relies on a clearance mechanism. On the one hand, central Aβ can be transported through the blood-brain barrier mediated by lipoprotein receptor-related protein and receptor for advanced glycation end products. On the other hand, Aβ can be degraded by insulin-degrading enzymes and neprilysin, or be bound by peripheral substances [28]. In addition, Aβ reuptake into neurons occurs in the presynaptic compartment [29].

### Distribution and Localization

Central Aβ is mainly produced in the brain. The cerebral cortex and hippocampus are believed to be regions that are enriched in Aβ and start their propagation. In AD brains, the Aβ deposits first appear in the neocortex, followed by allocortical regions, diencephalic nuclei, the striatum, and the cholinergic nuclei of the basal forebrain [60]; the entorhinal cortex is one of the most vulnerable regions [61]. Similarly, in normal brains, the cortex and hippocampus strongly express APP, suggesting the regions where Aβ abounds [62, 63].

The distribution of Aβ in subtypes of neural cells can be revealed by evidence of APP, and β- and γ-secretase. Although APP is widely expressed in a variety of tissues and cells, previous studies have shown that Aβ is more readily metabolized in neurons where BACE1 protein is abundant [38, 64], whereas other cell types mainly express BACE2, which is not involved in amyloidogenesis [65]. Similarly, neuronal APP has been identified as predominantly APP695 [66]. Besides neurons, glial cells, endothelial cells [67], and meninges [68] also express APP. Early studies showed that APPs in microglia and astrocyte were expressed in internal membranous vesicles [69] as isoforms containing Kunitz-type protease inhibitors [68, 70–73] rather than APP695. It was believed that the main source of Aβ was not glia cells but neurons [74], except for type I (GFAP+ A2B5–) astrocytes [75] or in a morbid environment [76]. Although this evidence is still *in vitro*, the contribution to the physiological Aβ biogenesis of astrocytes should be stressed since high levels of Aβ have been detected in human iPSC-derived astrocytes [77].

However, the specific neuron type that generates Aβ is still controversial. An immunocytochemical analysis has shown that APP is more frequently associated with glutamatergic rather than GABAergic or cholinergic terminals, indicating that endogenous Aβ is predominantly derived from excitatory neurons [78]. It has been reported that reducing neuronal activity using GABA-A receptor enhancers or increasing it with GABA-A channel blockers significantly reduces or increases Aβ levels (both Aβ40 and Aβ42) [6], stressing the contribution of GABAergic neurons to Aβ production. Given the high expression of APP in a heterogeneous subset of GABAergic interneurons, it has been reported that these interneurons take part in ~ 17% of the soluble Aβ and ~ 30% of the total hippocampal plaque burden, and interneurons are also located in the CA1 region, where plaques are most prevalent (accounting for ~ 75%) [79].

Within neurons, Aβ is located in neurites [80, 81]: biochemical, immunostaining, and electron microscopic studies have found APP and its fragments [66] in dendrites and axon terminals [24, 82]. Further, Aβ is supposed to be primarily released by synapses [83, 84]. It has been reported that Aβ levels in the brain interstitial fluid are considerably regulated by synaptic activity and synaptic vesicle exocytosis, implicating a mechanism on the presynaptic side of the synaptic cleft [51]. Notably, neuronal activity-dependent endocytosis of APP is involved in ~ 70% of the regulatory mechanisms in synaptic Aβ release [52].

In synapses, Aβ is predominantly distributed in the presynaptic membrane [78, 85]. Consistent with this, a meta-analysis of AD synaptic pathology showed that presynaptic markers are affected more than postsynaptic markers [86]. In normal or 5XFAD mice, BACE1 is localized to vesicles (possibly endosomes) at the ends of hippocampal mossy fibers, and in some cases, BACE1-positive vesicles are located near the synaptic active zone, suggesting Aβ production in the presynaptic membrane [87]. And it has been found that APP and BACE1 interact in biosynthesis and endocytosis, particularly along circulating microdomains such as dendritic spines and presynaptic boutons [88]. However, it has also been shown that in cultured murine neurons, γ-secretase is located both presynaptically and postsynaptically [89]. Furthermore, a recent super-resolution microscopy study found that co-labeling with APP is stronger postsynaptically than presynaptically [90]. Therefore, more evidence is needed to clarify the distribution of Aβ at the synapse.

### Dosage Effect

Under physiological conditions, the level of Aβ in the human brain and cerebrospinal fluid lies in the picomolar range [91, 92]. As the studied concentrations of Aβ42 ranged from femtomolar to millimolar, covering over twelve orders of magnitude [93], concentration matters for the physiological function of Aβ. According to the existing results, both too high and too low Aβ has negative effect on synaptic function, but only positive regulation has been reported in the physiological concentration range.

First, the detrimental effect of high levels of Aβ has been analyzed in the brains of AD and AD animal models. In these brains, Aβ concentrations tend to be in the nanomolar-to-micromolar range [94], which is much higher than the physiological level, thereby impairing synaptic function [95–97]. For example, the senile plaque requires a concentration of 100 nmol/L to aggregate Aβ, while Aβ42 is considered to gather when the concentration is up to 90 nmol/L [98]. Consequently, high concentrations of Aβ oligomers can cause the collapse of dendritic spines [84, 99] and disruption of LTP [100]. As the toxicity of pathologically overloaded Aβ is beyond the scope of this paper, further summary can be seen in reviews [14, 101]. Interestingly, inhibition of endogenous Aβ does not protect synaptic transmission as verso of a phenomenon in AD. The genetic knockout (KO) and pharmacological inhibition of Aβ production also have adverse effects on synapses. Varying degrees of cognitive deficits and synaptic damage are induced by knocking out the *APP* or *BACE1* gene, interfering with siRNA, or applying an inhibitor to wild-type mice, and interestingly, some evidence suggested this damage can be rescued by moderate amounts of Aβ (Table1).

Second, however, positive effects on synaptic regulation have gradually been discovered. Several studies have shown (Table1) that low concentrations (picomolar) of Aβ can enhance LTP [31], increase dendritic spine density [59], and promote docking vesicles [36]. The dose-dependence was demonstrated in an electrophysiological study at different concentrations (100, 200, and 300 pmol/L) of Aβ [49]. In another study, the full recovery of potentiation was at 300 pmol/L Aβ42, the threshold required for normal synaptic plasticity may be ~ 380 pmol/L [31]. Notably, the APP mutant A673T reduced Aβ by 40%–50% [102, 103] in a laboratory study and by ~ 28% in human plasma [104], which is thought to be protective against AD. Compared with mutations accelerating AD, the A673T mutation seems to reveal Aβ maintains a delicate balance to be a friend or foe in a dose-dependent manner.

Although the precise concentrations of Aβ to execute different acts in synaptic function are controversial, according to the available evidence, a “hormetic effect” seems to exist in Aβ roles: that is, a positive effect in the optimal dose range and a negative effect either above or below the range. The hormesis hypothesis may be a suitable explanation for the etiology of sporadic AD.

### Species Differences

Although it was recognized as early as the mid-1980s that Aβ is an endogenously-produced peptide, significant deposition of Aβ is often achieved by chimeras in animals with humanized mutations, so as to partially mimic the pathology of anthropic AD. However, the animal sequences of Aβ are distinct from humanized fragments to some extent. Blockade of endogenous Aβ with specific antibodies or ablation of APP expression impairs LTP and memory function [31]. Conversely, neurotransmitter release and recycling of synaptic vesicles are enhanced by increased endogenous Aβ1-40 or Aβ1-42 *via* interfering with clearance, or by applying picomolar amounts of synthetic fragments [53, 55, 105]. Therefore, the species differences in Aβ sequences matter as they function in synaptic regulation, although this seems to be complex.

On the one hand, 96.6% consistency has been identified between human and mouse APP, and only three amino-acid residues differ in the Aβ sequence [106]. However, endogenous picomolar Aβ does not induce Ca^2+^ homeostasis and synaptic integrity in neurons in mice, while high concentrations of Aβ from Tg2576 primary cortical neurons cause Ca^2+^ overload and synaptic damage [99]. This may be due to the sequence difference itself, or changes that occurred during biogenesis [107]. On the other hand, the impaired LTP, contextual fear memory, and reference memory induced by anti-rodent Aβ antibodies and siRNA against murine APP can be rescued by human Aβ42 [31]. Likewise, deletion of the *Drosophila* APP-like protein (Appl) is not lethal but has subtle behavioral defects that are partially rescued by expressing human APP [108]. Interestingly, the function of humanized APP varies with different mutations. Knockout of APP results in a significantly shorter body length and a short, curly tail in zebrafish. Wild-type human APP, rather than Swedish mutant APP, a mutation associated with familial AD, prevented these phenotypes [109]. In summary, the evidence suggests subtle relationships among Aβ sequences in various species, which needs more studies to clarify how much its functions are distinct or overlap.

### Isoforms and Aggregation

More than 20 forms of Aβ can be produced by enzymatic reactions and modification. Physiologically, Aβ40 is in the majority while Aβ37, Aβ38, Aβ39, and Aβ42 are in the minority, and peptides such as Aβ34, Aβ36, Aβ41, and Aβ43 are detectable in some instances [91, 110, 111]. Aβ segments are highly ordered, with 95% sequence identity between Aβ42 and Aβ40, except for a C-terminus of increased rigidity at Aβ42, which makes Aβ42 more prone to aggregation than Aβ40 [112]. As Aβ varies among monomers, oligomers, fibrils, and mature plaques, it remains difficult to identify the roles of endogenous pathological Aβ in AD patients. A widely held view is that Aβ oligomers, rather than fibrils or monomers, are the neurotoxic forms [100]. In the late 20th century and early 2000s, several studies showed that the soluble form of Aβ causes the loss of dendritic spines in cultured neurons, whereas fibrils and monomers are relatively inert [113–115]. Even at physiological concentrations, Aβ dimers, trimers, but not monomers, are deemed to cause synaptic dysfunction and loss [116]. However, the latest research on PS1 and PS2 conditional double-KO mice has shown that a reduced Aβ42 level is harmful to cognitive function, and the cognitive decline can be alleviated by giving exogenous soluble Aβ1-42 monomers [46].

In addition to the aggregated form, Aβ monomers themselves are also thought to have different functions. The hydrophobic C-terminal domains associated with oligomer formation are closely associated with neurotoxicity, especially at high levels (μmol/L) of Aβ. However, the hydrophilic N-terminal domain may mediate the protective action of Aβ at physiological levels (pmol/L–nmol/L). It has been found that the N-terminal Aβ fragment and shorter Aβ core (Aβ10–15) protect against or even reverse the effects of Aβ-induced neurotoxicity, memory deficits, and apoptosis [57]. Moreover, the rescue effect also occurs in 5XFAD mice and APP/PS1 mice, transgenic models of AD with significant Aβ deposition, especially at the level of synaptic plasticity [56]. Furthermore, a recent study has shown that fragments containing Aβ1–16 but not Aβ17–42 increase the size of the recycling pool of synaptic vesicles [54]. Therefore, it is critical to clarify the specific length of segments and aggregative form of Aβ *in vivo* when we explore its function.

## Potential Physiological Roles of Aβ in Regulating Synaptic Function

### Reduced Endogenous Aβ Impairs Synaptic Function

#### Blocking Aβ by Antibodies

The absence of Aβ appears to be detrimental to synapses. After antagonizing endogenous Aβ42 in rodents using JRF/rAb2 [31] or 4G8 [30], animals displayed cognitive deficits in behavior tests and impairment of LTP in electrophysiology. Moreover, injection of human Aβ42 rescued the above phenotypes, suggesting that endogenous Aβ plays a crucial role in normal LTP and memory. Abramov *et al*. further revealed a mechanism indicating that Aβ may positively regulate basal synaptic transmission in a presynaptic and history-dependent manner, particularly in excitatory neurons [55]. It was reported that a reduction in presynaptic strength by 53% ± 6% and inhibited exocytosis of synaptic vesicles occurred after using the monoclonal antibody HJ5.1 against murine Aβ, and this was reversible after a 30-min washout. In fact, the facilitation of vesicle release is diminished by both increasing and decreasing the endogenous extracellular Aβ concentrations. Similarly, short-term facilitation, which is believed to be closely related to memory formation, is not only impaired when Aβ excessively increases but when it dramatically decreases (> 60%), further suggesting that the action of Aβ exhibits dose-dependent [55].

Notably, Aβ is also thought to be involved in memory consolidation [30] and the forgetting mechanism by preventing subsequent modifications to provide adaptive physiological functions [45]. For instance, intracerebroventricular injection of 4G8 or knockdown of Fcgr2b, a receptor for soluble Aβ, regulates memory maintenance and forgetting in a novel object recognition test [117].

#### Deficiency or inhibition of APP

In early studies, APP-null mutant mice showed weight loss, abnormalities in locomotion, astrocyte gliosis at 14 weeks [32], and age-dependent cognitive deficits [33], with the cognition, altered weakly [34]. Recently, a reduction of LTP was reported when *APP* was knocked out or siRNA [31] interference was applied. In primary hippocampal neurons from *APP*-KO mice, there was synapse loss, restricted neurite growth, and reduced branching [35]. Interestingly, APP released by astrocytes was able to partially rescue this defect [118]. Furthermore, the absence of APP was shown to increase neuronal excitability. Although the two homologous analogues of APP, APLP1, and APLA2, do not produce Aβ, hippocampal neurons exhibit hyperexcitability when all three APP family genes are knocked out simultaneously in excitatory neurons [37]. Similarly, genetic loss of APP selectively impairs GABA-B receptor-mediated presynaptic inhibition and reduces axonal GABA-B receptor expression [119], indicating that this is a potential mechanism by which APP can regulate synaptic activity. The above studies that directly target APP somewhat of a contribution of Aβ to synaptic structural development and functions, but the role of APP itself should not be ignored.

#### Deficiency or Inhibition of BACE1

As a rate-limiting enzyme in Aβ processing, using BACE1 inhibitors seems to be a viable approach to attenuating Aβ and then benefiting AD. However, although significantly reducing Aβ production and amyloid deposition in the brain, BACE1 inhibitors can not improve the cognitive or functional decline in subjects with mild-to-moderate AD [120–122]. Compared to placebo, individuals who received a BACE1 inhibitor showed a dose-dependent cognitive deterioration and treatment-related adverse events, such as neuropsychiatric deficits and hippocampal volume loss in phase II and III clinical trials [123, 124], leading to the early termination of clinical trials. Interestingly, cognition returned to baseline levels after cessation of treatment [124]. This clinical evidence suggests that, at least in AD, remarkably reducing Aβ with a BACE1 inhibitor needs to be approached with prudence.

While clinical data are always limited to AD patients, pharmacological inhibition or genetic modification of BACE1 in wild-type mice can partly reveal the physiological roles of Aβ. In animal experiments, gavage of the blood-brain-barrier-permeable BACE1 inhibitors Verubecestat (MK-8931) and Lanabecestat (AZD3293) to mice resulted in a dose-dependent decrease in LTP [125]. Oral administration of the BACE1 inhibitors SCH1682496 or LY2811376 also caused a dose-dependent decrease in Aβ40 levels, but prolonged treatment suppressed dendritic spine formation in layer V pyramidal neurons, which recovered after drug discontinuation [44]. Consistent with this, BACE1-deficient mice exhibit impaired synaptic transmission and plasticity, evidenced by reduced LTP in Schaffer collateral branch-to-CA1 synapses and mossy fiber-to-CA3 synapses [41, 43]. In another study, deficits in paired-pulse facilitation and de-depression implicated in presynaptic release and synaptic plasticity were recorded in *BACE1(-/-)* mice, and the poor performance on tests of cognition was prevented by APP/PS1 transgenic mice [38]. Moreover, it has been suggested that inhibition or deficiency of BACE1 leads to reduced docking of synaptic vesicles to the active zone and the ensuing glutamate release [125]. To avoid the developmentally-relevant phenotypes in germline mutant mice, researchers have turned to conditional KO of exon 2 of *BACE1* in adult mice, in which impairment of synaptic and axonal function also occurs [39, 126]. For example, in BACE1^fl/fl^; R26CreERT2-TAM mice, BACE1 is reduced by 90%–95%. and Aβ is inhibited by ~ 60%–90%, followed by axonal dysfunction [126]. Collectively, the failure of BACE1 inhibitors, which cause a strong reduction in Aβ deposition, may largely be due to their role in synaptic function.

Given the harmful effects associated with synaptic damage, it appears that complete or significant inhibition of BACE1 neutralizes or even overwhelms the anticipated therapeutic effect against an Aβ burden. Both clinical [123, 124] and laboratory [125] results have confirmed that this impairment is dose-dependent; in addition, this can be partly explained by the fact that germline heterozygous BACE1-KO mice with 50% of normal BACE1 levels do not differ significantly from wild-type mice [38, 127, 128]. Therefore, the dosage is vital. Admittedly, however, seizures [129, 130], axon guidance [131], impaired peripheral nerve myelination [132, 133], and low anxiety or depressive tendencies [38] have been sequentially reported in BACE1-null mice. All these side-effects are consistent with adverse events in clinical trials with BACE1 inhibitors [123, 124], although some of the phenotypes remain controversial [42]. This indicates that the functions of BACE1 itself should be taken into account.

Collectively, Aβ is necessary for maintaining the normal synaptic function, reduced endogenous Aβ by genetic or pharmaceutical inhibition of Aβ or its biogenic necessities, APP and BACE1, disturb synaptic morphology, synaptic vesicle transmission, synaptic plasticity, and even cognitive function. Although several studies have revealed a rescue effect [30, 31, 47] and implied a dosage effect, more detailed and persuasive results are needed to draw firmer conclusions.

### Interplay: Aβ and Neural Hyperexcitability

#### Moderately Increased AΒ Enhances LTP and Neuronal Excitability

As previously noted, the body produces Aβ endogenously at picomolar concentrations, and either too low or too high Aβ may impair synaptic function. However, a number of early and recent studies have demonstrated that a modest increase of Aβ can enhance synaptic transmission and neuronal excitability, providing further evidence for its physiological function. Puzzo *et al*. have worked long on the role of Aβ in physiological states, particularly in synaptic regulation and cognitive function. They initially administered intrahippocampal injections or delivered picomolar levels of Aβ42 to mouse brain slices, and found that Aβ42 enhanced LTP and behavior performance [50]. Given the toxic effects of excess Aβ42 in AD, the team investigated the LTP variation with different concentrations of Aβ42 in order to clarify the dose-effect relationship, which finally took on a bell-shaped curve [49]. Besides, the exposure time also matters [48]. Furthermore, by inhibiting thiorphan, an enzyme degrading Aβ in the synaptic cleft, the acute effects of endogenously-released Aβ were investigated at single presynaptic terminals and synaptic connections [53, 55]. These studies demonstrated that Aβ mediates presynaptic enhancement and synaptic transmission by increasing miniature synaptic vesicle release and mEPSC frequency, which depends on the history of activation. As deprivation of endogenous Aβ reduces presynaptic activity, it has been speculated that Aβ maintains basal presynaptic activity and spontaneous activity [55]. In recent years, more studies have focused on the aggregated forms and effective sites of Aβ. So, several studies have shown that it is the N-terminal Aβ, particularly the 1–16 fragment, that exerts excitatory effects and promotes vesicular recycling [54, 58], even reversing the Aβ toxicity. Besides, Aβ42 oligomers, commonly regarded as toxic, have been reported to enhance synaptic plasticity at picomolar concentrations [36, 46, 47].

Since the direct application of soluble Aβ in wild-type mice increases neuronal activation [134], what is the situation in early AD or AD model mice with a mild to moderate increase in Aβ?

*In vivo* Ca^2+^ imaging of somatic, dendritic, and axonal activity patterns in cortical neurons has shown that both healthy ageing and AD-related mutations have neuronal hyperactivity [135]. In the hippocampus of young AD model mice, hyperexcitable neurons are selectively increased prior to plaque formation. In these animal models, acute treatment with the γ-secretase inhibitor LY-411575 reduces soluble Aβ levels and rescues the neuronal dysfunction, while administration of soluble Aβ oligomers re-establishes the excitatory state [134]. Here, soluble forms rather than aggregates matter. However, in the AD mouse model, two-photon data displayed that not all neuronal activity is reduced or increased, and it is in the vicinity of plaques where part of the neurons with hyperactivity are exclusively found [136, 137]. The mechanism is attributed to the fact that low levels of Aβ enhance glutamate release and regulate Ca^2+^ homeostasis, particularly in the early stages of AD [99].

Aβ is altered 20–25 years prior to the onset of AD [138, 139]. Individuals at risk for AD always manifest hyperactivation in memory-related brain regions in functional magnetic resonance imaging (fMRI). APOE (apolipoprotein E) ε4 carriers at 25–35 years old present increased co-activation of the default mode network and a more activated hippocampus during encoding tasks compared to non-carriers in fMRI studies [140]. Another study not only found that cognitively normal APOE ε4 allele carriers have a greater magnitude and greater extent of brain activation than APOE ε3 allele carriers during a memory activation task but also showed that the extent of baseline brain activation correlated with the degree of memory decline after a 2-year longitudinal assessment [141]. In addition, young subjects with normal cognition who carry the familial AD gene *E280A PS1* mutation, have hippocampal activation before the onset of symptoms at ~ 45 years old [142]. The same phenomenon has been demonstrated in patients with amnestic mild cognitive impairment (aMCI) [143–146]. Therefore, increased Aβ at an early stage may take part in the regulation of cognitive function, possibly by inducing synaptic dysfunction, but the underlying mechanisms remain to be solved.

#### Neural Hyperexcitability Promotes Aβ Production

Endogenous Aβ increases neuronal excitability [147], while neural activity also regulates Aβ production. Laboratory studies have shown that neuronal and synaptic activity dynamically regulates soluble extracellular Aβ concentrations [6, 31, 148]. The rapid effects (a timescale of minutes to hours) of synaptic activity on Aβ were investigated by microdialysis combined with field potential recordings, in which it was demonstrated that synaptic activity dynamically and directly regulated Aβ in the brain interstitial fluid (ISF) [51]. Further, ISF Aβ levels were elevated by enhancing synaptic transmission and were prevented by inhibiting endocytosis mediated by clathrin. The above evidence suggests that Aβ release depends on synaptic activity mediated by endocytosis [52].

Furthermore, clinical phenomena abound suggesting that alterations of brain activity are accompanied by changes in Aβ level. The regions active in the default state in young adults have a higher propensity for Aβ deposition in the old with AD [149]. In addition, evidence from epilepsy and post-traumatic states provide a good illustration. First, patients with epilepsy, particularly late-onset epilepsy of unknown etiology, are at higher risk of developing dementia. Simultaneously, seizures have been detected in the early stages of AD [150]. As previously noted, because patients with aMCI exhibit elevated hippocampal activation in the dentate gyrus or CA3 region, Bakker *et al*. [151] reduced hippocampal hyperactivity in aMCI with the antiepileptic drug levetiracetam, and, as expected, cognitive function was improved. Second, the increased ISF Aβ in 18 patients with acute brain injury showed a strong positive correlation between Aβ level in the ISF and neurological status [152]. The fact that ISF Aβ varies along with neuronal function further implies that the extracellular Aβ level is regulated by neuronal activity.

### Underlying Mechanisms in the Regulation of Aβ at Synapses

#### nAChR

In the central nervous system, the nicotinic acetylcholine receptors (nAChRs) are located at synapses in most neuron populations [153] as well as being expressed in non-neuronal cells [154–156]. nAChRs are ligand-gated ion channels. Depolarization of the membrane and excitatory effects are caused by the application of nAChR agonists followed by opening ion channels, and consequently, increasing permeability to Na^+^/K^+^/Ca^2+^. The α7 nAChR has the highest Ca^2+^ permeability among nAChR isoforms, so its relative permeability is comparable to that of the N-methyl-D-aspartate receptor (NMDAR) [157]. Overall, α7 nAChRs are involved in a variety of biological processes, including neuronal excitability, neurotransmitter release, signal transduction, synaptic plasticity, and neurogenesis [158–160].

In the brains of AD patients, the reduction of nAChRs is correlated with disease progression [161, 162], and cholinesterase inhibitors are widely used in the treatment of mild to moderate AD. α7 nAChRs have been found to co-localize with intracellular Aβ42-positive neurons in the post-mortem brain tissue of AD patients [163]. Similarly, an increase of Aβ/nAChR-like complexes has been found in carriers of APOE ε4 [164], a strong risk factor for AD [165]. In fact, nAChRs interact with Aβ under physiological conditions, particularly the α7 isoform, which has a high affinity for Aβ [166, 167]. Furthermore, 12-month-old α7 KO mice exhibit an AD-like pathology, in which elevated Aβ is thought to be a compensatory response to the deletion of nAChRs [168]. It has been reported that low levels of Aβ (picomolar the low nanomolar range) activate α7 nAChR channels [50, 169] possibly *via* the nitric oxide/cGMP/protein kinase G pathway [36]. In contrast, higher levels (nanomolar the low micromolar range) reduce the duration of ACh-induced activation [170], leading to dysregulation of electrical activity at synapses [171]. However, controversially, nicotine, another ligand of nAChRs, is reported to improve cognition and protect neurons from Aβ damage by agonizing nAChRs [172, 173]. This paradox has been explained by the suggestion that different cellular pathways and downstream mechanisms are initiated. That is, nicotine acts through PI3K–AKT, JAK–2/STAT-3, and other mechanisms to exert protective effects, whereas Aβ is thought to initiate intracellular signaling cascades like the MAPK kinase pathway and leads to cell death [174].

In addition, nAChR subtypes other than α7 participate the synaptic mechanism of Aβ. For instance, α7β2, a variant of α7, is more sensitive to pathological concentrations of Aβ [175]; mice with β2nAChR deletion display neurodegeneration [176] despite the amelioration of spatial reference memory in APP/PS1 mice by β2 deficiency [177]; and α4β2 nAChR is particularly associated with episodic memory and working memory [178], while selective co-activation of α7 and α4β2 nAChRs is also sufficient to reverse Aβ-induced AMPA receptor dysfunction and LTP alterations. Due to the structural differences of nAChRs [179], Aβ might interact with specific subtypes to varying degrees. Aβ and nAChRs form complexes through multiple sites [180] to mediate the physiological effects of Aβ or toxicity to cholinergic neurons. For example, when cell lines expressing α4β2 nAChRs are exposed to nanomolar Aβ 42, the expression of genes related to Ca^2+^ signaling and axonal vesicle transport is upregulated while genes related to metabolic, apoptotic, or DNA repair pathways are downregulated [181]. Notably, the results did not mimic physiological stations because the high concentration of Aβ and overexpressed nAChR receptors were used in this research.

The complexity of relationships between Aβ and nAChRs is evident, but the dose of Aβ applied and the aggregation state still need to be considered [56, 182]. Although nAChRs are weakly expressed in AD, they maintain normal or even increased mRNA (for review see [174]). Besides, the extreme susceptibility of nAChRs to desensitization may partially explain the paradox between nicotine and Aβ, or even the variation of the Aβ dosage effect.

#### The N-methyl-D-aspartate Receptor

The NMDAR belongs to the ionotropic glutamate receptor family, and it enhances synaptic transmission and plasticity [183] mediated by Ca^2+^/calmodulin-dependent protein kinase II [184, 185], which triggers a signaling cascade. The NMDAR has been found to be critical for neurons [186, 187]. Antagonism of NMDARs gives rise to apoptosis and degeneration, while moderate activation of this receptor benefits neuron survival; however, the excessive activation of NMDARs causes Ca^2+^ overload, resulting in excitotoxicity. Therefore, both inactivation and overactivation are potentially harmful [188, 189]. In addition, it has recently been suggested that synaptic NMDARs and extrasynaptic NMDARs play very different roles [190, 191], where the former is thought to be beneficial and the latter to mediate toxic effects [183, 192–195].

Accumulation of Aβ oligomers has been observed in the synapses of glutamatergic neurons in AD brains [196, 197]. Although plenty of studies have demonstrated that Aβ mediates neurotoxicity by directly or indirectly regulating NMDARs [6, 198–202], and NMDAR antagonists can rescue Aβ-induced damage [116, 203], interestingly, genetic deletion of the NMDAR subunit GluN3A results in neuropathological changes like AD, including psychological/cognitive deficits and amyloid-β/tau pathology [204]. Moreover, blocking NMDARs may reduce neurodegeneration [205]. Therefore, as a non-competitive, specific, low-affinity NMDAR antagonist with a fast closing rate, memantine is used to treat moderate to severe AD [206] since it can reduce excitotoxicity while preserving normal NMDAR activity at the same time. Notably, it has been shown that memantine preferentially targets the extrasynaptic NMDAR [193] which is regarded as a detrimental characteristic of AD.

The multiple possibilities for NMDARs in terms of dose, subunit type, and subcellular localization make research on the relationship between Aβ and NMDARs difficult. Complete inhibition, low to mild activation, and over-activation have dramatically distinct effects. Besides, different subunits vary: for example, GluR2A and GluR2B each interact with Aβ to cause opposite results [207]. Moreover, careful investigation is needed, for example, on the roles of D-serine and glycine, co-agonists of synaptic NMDARs and extrasynaptic NMDARs, respectively [208]; which downstream pathways are activated by NMDARs with different subcellular localizations; whether or not NMDARs are translocated on the cell membrane.

#### Vesicular Circulation

The synaptic vesicle cycle (SVC), comprising vesicle trafficking, docking, fusion, transmitter release, and regeneration of fresh vesicles [29], plays a crucial role in the biology of synaptic terminals by way of recurrent exocytosis and endocytosis [209]. Due to the strong positive correlation between cognitive decline and synaptic loss [13, 210], research on synapses exposed to Aβ is increasing [14, 211]. A convergence of results points out a reciprocal relationship between Aβ and the SVC. For one thing, Aβ regulates the SVC *via* dosage effect, sites of action (pre- and post-synaptic), and pattern of action (local autocrine or paracrine), for another, the SVC also affects the production of Aβ [29].

Studies so far suggest that the SVC can be regulated by Aβ. The absence of Aβ impairs vesicular docking in active zones [125]. Picomolar or low levels [28] of Aβ have been shown to enhance synaptic transmission by upregulating the presynaptic neurotransmitter release probability (Pr) [55]. Furthermore, Lazarevic *et al*. systematically studied the concentration effect in Aβ regulation at the synapse. There, they found that the SVC decreases when Aβ is depleted by modulating production while the SVC increases by using an endogenous Aβ degradation inhibitor [53, 54].

On the contrary, a high level of Aβ inhibits Pr [96]. Either natural or synthetic Aβ oligomers but not monomers [116] at high doses inhibit synaptic transmission and plasticity [212–214]. Intracellular administration of nanomolar Aβ42 significantly cuts down LTP, reduces mEPSC amplitude, and decreases the number of intrasynaptic vesicles and/or Pr [215]. Moreover, direct injection of Aβ42 oligomers into presynaptic axon terminals results in a blockade of synaptic transmission [216], and even acute exposure to Aβ oligomers reduces postsynaptic current frequency by ~ 50% [116]. A series of studies have proposed that Aβ is involved in many steps of the SVC. First, Aβ perturbs the formation of fusion complexes, as reported in postmortem AD brains, where the SNARE complex, which is essential in driving synaptic vesicle fusion in the presynaptic active zone, is significantly reduced [217]. Second, the interaction of SNARE protein vesicle-associated protein 2 (VAMP2) with synaptophysin is necessary and sufficient to recruit VAMP2 to synaptic contacts, and it is disrupted by internalized Aβ42 [218]. Further, the ability of clathrin-dependent endocytosis is a critical step in the SVC, and a wealth of evidence, including genomics and proteomics, shows that such endocytosis is severely disturbed in AD [219–222]. In Aβ oligomer-treated neurons, only 50% of the released vesicles are recycled back in time, leading to a considerable delay in readily-releasable pool recovery [223]. In particular, atypical cyclin-dependent kinase 5 (CDK5) [224] plays a major role in regulating the size of the synaptic vesicle pool by targeting synaptic vesicle endocytosis [225], and consistently, CDK5 is significantly higher in postmortem AD brains [226, 227]. Although substantial studies have been devoted to the mechanisms of Aβ toxicity, from another perspective, some of these results also imply that Aβ is a potent target for presynaptic regulation both in physiology and pathology. Together, endogenously released Aβ peptides are crucial for maintaining a normal SVC in the functional range.

*Vice versa*, the SVC takes part in Aβ production [22, 23]. Indeed, non-amyloid cleavage of APP occurs on the cell membrane, while amyloid cleavage of APP by β- and γ-secretase is facilitated in vesicles, leading to Aβ production and release [19]. This process has been shown to be upregulated by increased neuronal activity and clathrin-dependent endocytosis [51, 52], thereby promoting Aβ production and even affecting the ratio of Aβ42 to Aβ40 [55].

#### Other Mechanisms

Other than the above mechanisms, glial cells, energy metabolism, and other factors participate in the role of Aβ in synapses through direct or indirect regulation. (1) Microglia. On the one hand, microglia can be activated by Aβ and then mediate synapse pruning and elimination [228]; on the other hand, the activated immune system influences the aggregation of Aβ to cause diverse effects [229] (e.g. microglia secrete galectin3 to promote the oligomerization of Aβ [230]). (2) Astrocytes. Astrocytes participate in the processes of synapse engulfment [231], besides which, they secrets APOE, a risk factor of sporadic AD, to result in synaptic degeneration by enhancing the abnormal aggregation of Aβ at synapses [232, 233]. (3) Energy metabolism. The synapse is vulnerable to energy deficiency as a highly energy-consuming structure, especially in vesicle cycling [12, 234]. It has been shown that subthreshold amyloid deposition or the distribution of Aβ is correlated with increased aerobic glycolysis in early adulthood [235–237], whereas aerobic glycolysis decreases in the normal aging brain [238].

## Perspectives

The underlying role of Aβ in regulating synaptic functions seems to have being revealed gradually. However, several key limitations need to be noted. (1) Animal models. Although diverse animal models have been developed to study AD and Aβ, most of them are genetically-manipulated mice carrying mutations of human familial AD [106, 239–241]. Species differences should be taken into account when they poorly mimic the pathological process of human AD. In fact, human-derived Aβ fragments are more likely to be deposited, and hAPP transgenic mice without expression of endogenous murine APP display more plaques and faster Aβ deposition [230, 242]. (2) The role of APP or BACE1. APP performs its functions concurrently with its various products including Aβ, all of which also join in the regulation of synapses. For example, sAPP contributes to synaptic function [243, 244], partly as a ligand to regulate synaptic transmission [245]. Possibly, Aβ does not act alone [246]. Similarly for BACE1, in models with BACE1 deletion, impaired axonal guidance is associated with reduced hydrolysis of CHL1 (cell adhesion molecule L1-like) [131], and synaptic damage is associated with seizure protein 6 [247], both of which are substrates of BACE1. (3) The complexity of Aβ itself. All the following factors matter in Aβ functions: length of fragments [248], concentration, intracellular or extracellular localization, and aggregation state [47]. For instance, nanomolar concentrations of intra-axonal oligomeric Aβ42 (o Aβ42), but not oAβ40 or extracellular oAβ42, acutely inhibit synaptic transmission in squid [6]. Besides, the effect of Aβ varies with time under both physiological [48] and pathological conditions [249]. Population studies have shown that Aβ deposition does not increase all the time, while CSF Aβ42 is significantly negatively correlated with disease progression [139]. All the above reveals that Aβ plays different roles through a dynamic balance of time and state. Generally, limited by the complicated biophysical characteristics of Aβ aggregation [250], the state of Aβ in the laboratory is not always comparable with that *in vivo*. Therefore, research on the physiological mechanisms of Aβ still needs a more rigorous and unified paradigm.

In conclusion, although the history of research on the mechanism of Aβ is long, the role of Aβ itself under physiological conditions is still poorly understood. Successive failure in clinical trials has brought investigators back to the original and intrinsic question: what is the physiological role of Aβ ? Undeniable evidence has established that Aβ plays a key role in AD, and this appears to imply an equally important role in physiological memory regulation. As an essential structural base of memory formation, the synapse is a promising target for research. However, research is difficult because of the above challenges. In the future, we should design effective approaches to imitate the physiological Aβ environment as much as possible, and more animal models with increased homologous Aβ should be developed, to reveal the physiology and understand the pathology in AD.
